# Psychological and Psychosocial Consequences of Zoonotic Cutaneous Leishmaniasis among Women in Tunisia: Preliminary Findings from an Exploratory Study

**DOI:** 10.1371/journal.pntd.0005090

**Published:** 2016-10-27

**Authors:** Mohamed Kouni Chahed, Hédia Bellali, Sonia Ben Jemaa, Tarek Bellaj

**Affiliations:** 1 Department of Epidemiology and Public Health, Faculty of Medicine of Tunis, Tunis-El Manar University, Tunis, Tunisia; 2 Research Unit “Analysis of the effects of environmental and climate changes on health”, Department of Epidemiology and Statistics, A. Mami Hospital, Ariana, Tunisia; 3 Psychology Department, University of human sciences of Tunis, Tunis, Tunisia; 4 Social Sciences Department, College of Arts and Sciences, Qatar University, Doha, Qatar; University of Notre Dame, UNITED STATES

## Abstract

**Background:**

The incidence of zoonotic cutaneous leishmaniasis (ZCL) makes it the most widespread parasitic disease in Tunisia and the Arab world. Yet, few studies have addressed its psychological and psychosocial effects. The purpose of this study was to examine the psychosocial impact of ZCL scars among Tunisian women.

**Methods:**

We conducted an exploratory study, we administered Revised Illness Perception Questionnaire (IPQ-R), World Health Organization Quality Of Life-26 (WHOQOL-26) and Psoriasis Life Stress Inventory (PLSI) to a group of girls and women with ZCL scar in the region of Sidi Bouzid. This group was randomly selected from volunteers who came to primary health care facilities to seek for treatment for any pathology.

**Results:**

Descriptive statistics showed that the collected scores from the three scales exhibit heterogeneous distributions: IPQ-R (M = 63.6, SD = 15.6), PSLI (M = 9.5, SD = 6.7), WHOQOL-Physical (M = 63, SD = 12.9), WHOQOL-Psychological (M = 52.6, SD = 11.1), WHOQOL-Social (M = 61.8, SD = 17.5), and WHOQOL-Environmental (M = 47.8, SD = 13.3). The correlation analyses performed on Inter and intra-subscales showed that the emotional representations associated with ZCL were correlated with the loss of self-esteem and feelings of inferiority (r = 0.77, p<0.05). In addition, high education level and the knowledge about ZCL are positively correlated with cognitive and emotional representation in the IPQ-R (r = 0.33, p<0.05). "Rejection experiences" and the "anticipation and avoidance of stress" were respectively negatively correlated with age (r = -0.33, p<0.05 and r = -0.31, p<0.05). Correlations between the scores on IPQ-R domains and PLSI factors were significant. The results showed that anticipation of rejection and avoidance of stress are strongly correlated with a negative perception of ZCL. Quality of life scores were not correlated with either age, education level, time of illness, or the number of facial or body scars. However, the correlations between quality of life scores and the multiple IPQ-R domains were all insignificant. Finally, there was a negative correlation between the scores on the perceived quality of social life and the knowledge about ZCL (r = -0.34, p<0.05).

**Conclusions:**

This makes it vital to strengthen preventive health education. Conducting studies on ways to establish a holistic support system for managing ZCL, a system that covers the psychological challenges and the barriers it causes to women’s social and professional integration, is a vital first step.

## Author Summary

Zoonotic cutaneous leishmaniasis (ZCL) is the most common form of leishmaniasis in Tunisia. The disease is not severe and heal spontaneously with a definitive scar causing a social impact mainly when the lesion occurs in the face. Yet, few studies have addressed these psychological and psychosocial effects. To examine these issues among Tunisian women suffering from ZCL, we administered Revised Illness Perception Questionnaire (IPQ-R), World Health Organization Quality Of Life-26 (WHOQOL-26) and Psoriasis Life Stress Inventory (PLSI) to a group of girls and women with ZCL scars in the region of Sidi Bouzid. This study demonstrated the wide range of psychological effects (anxiety, psychological distress, lack of self-confidence and self-esteem, frustration, etc…) and psychosocial impacts (stigma, rejection, discrimination in the social and professional setting, etc…) of ZCL permanent scars; mainly when it is located in the face; among women and girls. This study suggests the need of social and psychological support and professional integration for women and girls in rural remote areas first and second conducts studies on ways to establish control measures for ZCL, mainly in vulnerable exposed population.

## Introduction

Cutaneous leishmaniasis is the most common form of leishmaniasis. It is a skin infection caused by a unicellular parasite transmitted by infected sandflies that feed on the blood of rodents bred in caves or burrows in degraded environments. It is known also as Oriental Sore, Biskra Button, Aleppo boil or Tabaa Sidi Bouzid, depending on the geographic region concerned.

Cutaneous leishmaniasis is endemic in many parts of the world. There are about twenty different species of Leishmania capable of infecting humans. The distribution of cutaneous leishmaniasis is very closely related to the geographical characteristics and the ecological specificities of the endemic areas.

Cutaneous leishmaniasis always heals spontaneously, but leaves permanent scars. New treatment options and new molecules are still in the process of getting validated. ZCL is the most widespread parasitic disease in Tunisia. It mostly affects people who live in the central and south-western parts of the country.

ZCL is not a fatal disease, but it causes significant changes in victims that affect their psychosocial condition and quality of life. Work done in Pakistan and Afghanistan [1] suggests that the level of stigma and social exclusion suffered by ZCL victims is tied to the number and visibility of their ZCL scars. Affected girls and women have fewer chances of getting a job, getting married and leading a fulfilling social life [2]. This shows that ZCL can have severe consequences in many respects, for it seriously restricts the subject’s social, economic and cultural life.

The theoretical framework for this study is the self-regulatory theory framework by which Leventhal, Meyer & Nerenz [3] explain adaptive behaviour during a health problem. In the self-regulatory model [4], illness perception and coping style are directly related. A person’s perception of illness contributes considerably to guide their choice of coping strategies.

According to Leventhal, Nerenz and Steele [4], there are five domains of illness perception. These are *identity*: what the illness is called and the symptoms that affect the patient and are associated with the illness; *the cause*: the patient’s opinion on the causes of the illness. An illness with no identifiable cause is a devastating experience that causes impotence and difficult adjustment; *the timeline*: chronic, acute or cyclical; *the consequences*: for the patient and his entourage; and *cure and control*: this domain refers to personal perceptions of one’s potential power to control the illness and its spontaneous development or treatment.

Apart from these five domains of illness perception, there are other domains such as *emotional representation*, relating to all the negative feelings caused by the illness or *the coherence of the illness*, a metacognitive domain relating to the general understanding of the illness and to the degree of integration of its various dimensions, symptoms and causes.

Our study intended to evaluate the psychological and social impact of ZCL on women with facial scars in the region of Sidi Bouzid. Understanding perceptions of ZCL and quality of life among patients with ZCL and ZCL scars can help us develop psychosocial intervention strategies to fight against stigma and self-deprecation, and to develop strategies for preventing harmful behaviour patterns on ZCL.

## Methods

### Study design and population

**Fig 1:**
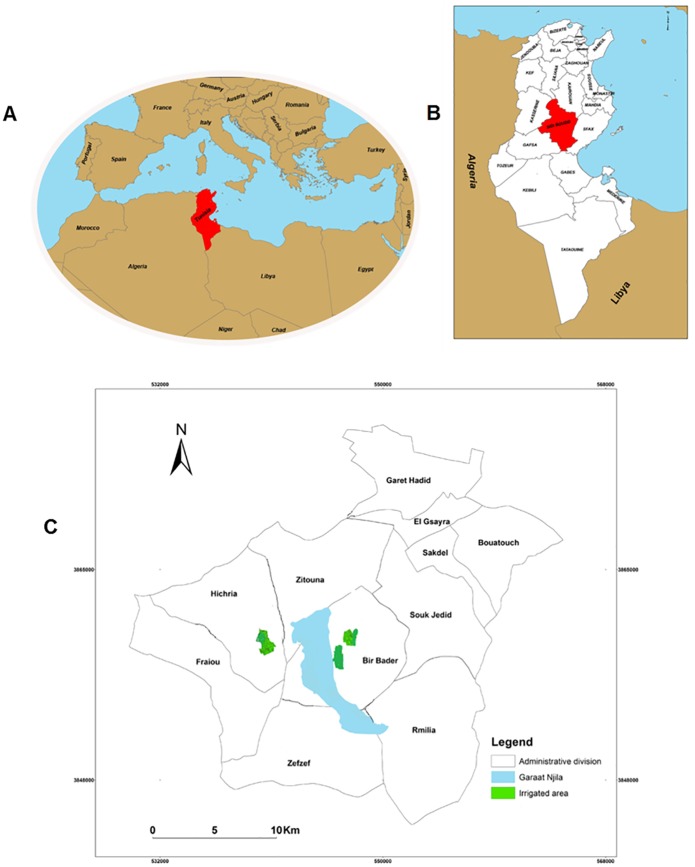
Study area. (A) Location of Tunisia within the Mediterranean basin, (B) Location of Sidi Bouzid gouvernorate within Tunisia, (C) Location of the study areas (Bir Badr and Hichria).

The study was conducted in Sidi Bouzid, a predominantly rural or semi-rural region with scattered groups of extended families attached to their farmland. Sidi Bouzid is a region that provides over 20% of the country's agricultural production. There is no psychological services available for this population. For the facility and the feasibility of this first study and the disponibility of the population, the sample was made up of 41 girls and women from El Hichria (n = 31) and Ouled Mhamed (n = 10) in the Sidi Bouzid Governorate (Fig 1). We conducted an exploratory study among a volunteer group of girls and women selected randomly in primary health care facilities during their medical visits for any pathology or for kids routine vaccination. This study focused on the impact of ZCL scar among females because they are most vulnerable to the psychosocial consequences than males. They had facial and non facial scars due to ZCL and were relatively young, the age ranged from 12 to 53 years old (85% are under 30 years). Ninety three per cent of the respondents had at least one facial scar and 54% had scars on other parts of the body.

### Study tools

#### Evaluating illness perception: The IPQ-R

To assess the perception of health and illness, we used the "Revised Illness Perception Questionnaire (IPQ-R [5])." This is the most commonly used generic illness perception questionnaire in health psychology. The questionnaire was translated and adapted to the specific requirements of ZCL as skin disease, as well as to the idiosyncratic cultural characteristics of Sidi Bouzid. After the tool was translated, back-translated and adapted, it was pre-tested together with primary healthcare providers in Sidi Bouzid to make the necessary final adjustments.

The IPQ-R contains 71 questions rated on a four-level Likert scale on level of agreement with the item (strongly disagree, disagree, agree, strongly agree). It explores the illness identity, timeline, consequences, cure, control, and coherence domains and the emotional representation domain.

During scaling and scoring, we did reverse recoding of several items. The results for the domains are expressed in percentages.

#### Assessing psychosocial adjustment to stress from skin disease

The assessment of the psychosocial impact of leishmaniasis was done using the Psoriasis Life Stress Inventory (PSLI) questionnaire [6]. The questionnaire, designed initially for patients with psoriasis, can be used for other skin diseases and is translated, back-translated and adapted, in this study, for use on patients with ZCL. For each of the 15 items of the PLSI, the patient shows how each element of stress was experienced over the last 4 weeks on a four-level Likert scale: from "not at all" (score 0) to "very much" (score 3). The total score of stress may therefore vary from 0 to 45. The questionnaire therefore provides a general assessment of psychosocial stress experienced on a daily basis after getting a skin disease. It describes the impact of a skin disease, such as ZCL, on the lives of patients, including their emotional and social lives, and focuses on quantifying the stress experienced by ZCL patients and the role of stress factors in their adjustment.

#### Assessing quality of life: WHOQOL-26

To assess the quality of life of adolescents and women affected by ZCL, we chose to use the World Health Organization Quality Of Life-26 (WHOQOL-26) scale [7]. This scale assesses a subject’s general state of health by understanding their physical and mental state, and by assessing the changes in their work, relationships and social activities. It was developed collaboratively by WHO to serve as an international cross-culturally comparable instrument.

Four major factors of quality of life were examined to enable us to have a sufficiently comprehensive perception of the perceived quality of life of the respondents according to four domains: 1. The physical domain, which covers items on physical pain feeling, the need for any medical treatment, energy for everyday life, sleep satisfaction, functional capacity, energy and work capacity; 2. The psychological domain, which contains items exploring aspects of perceived satisfaction about the meaning of life, the capacity of concentration, the bodily appearance, self-image, and negative feelings; 3. The domain of social relationships, which includes items related to satisfaction about personal relationships, sex life, and social support; 4. The environmental health domain, which comprises items related to perceived safety in daily life, financial resources, information availability, leisure activities, condition of living place, accessibility to health services, and transportation.

### Study procedure

The questionnaires were administered in the following order: the IPQ-R, the PLSI and WHOQOL Bref.

The information gathered was processed using *Statistica 6* software after verifying that it was entered properly. We performed statistical analysis using parametric tests after checking for the normal distribution of variables. The correlation analysis were done using the Pearson correlation coefficient. For comparison, we used *Student’s t*-test or variance analysis. We didn’t perform multivariate analysis because of the small sample size.

### Ethics statement

The questionnaires were administered by a psychologist and/or a health educator on ZCL patients in their homes or at the local clinic. All persons who participated in this study did so willingly. The study was approved by the "Ethic committee of Pasteur Institute of Tunis (IPT)".

All women provided informed consent, and parents of any child participant provided also informed consent on their behalf. The informed consent given by all subjects was oral since the majority of them were illiterate, the psychologist and the health educator explained the aim and the procedure of the study and asked them for consent. The "Ethic committee" approved the study since we didn't need any clinic or biological investigation from all subjects with the guarantee from the research team about confidentiality and the respect of individual information. The response on the questionnaire attest the oral consent of participants.

## Results

Table 1 illustrate the distribution of scores on the different scales. It showed that the collected scores from the three scales exhibit heterogeneous distributions especially for the PSLI (Mean: 9.5, SD, 6.7 and Coefficient of variance: 70.7) whereas for all the other variables coefficient of variance did not exceed 28.3. It suggests that level of psychosocial adjustment to stress from CL is variable across the participants.

**Table 1:** Distribution of the scores on the IPQ-R, PSLI and Whoqol different domains.

	Mean	Min	Max	Std.Dev	Coef.Var.	Skewness	Kurtosis
IPQ-R	63.6	34.0	104.0	15.6	24.5	0.6	0.2
PSLI	9.5	0.0	25.0	6.7	70.7	0.6	-0.3
Whoqol domains							
Physical	63.0	28.6	82.1	12.9	20.4	-0.8	0.5
Psychological	52.6	29.2	79.2	11.1	21.0	0.2	-0.2
Social	61.8	12.5	100.0	17.5	28.3	-0.7	0.7
Environmental	47.8	18.8	75.0	13.3	27.9	-0.5	0.1

Min: Minimum, Max: Maximum, Std.Dev: Standard deviation, Coef.Var: Coefficient of variance.

### Perception of cutaneous leishmaniasis

For the respondents in the survey, this disease, "leishmaniasis", is linked with sand flies. All the respondents stated that they consider ZCL scars as marks of the disease, and that the number of scars varies from person to person. Seventy-eight per cent considered that ZCL is sex-specific. Seventy-eight per cent declared that it is seasonal and spares no age group. Seventy per cent believed the insect bites eventually become scars. The respondents had different views on whether this disease was found recently (56%) or a long time ago and on the possible disappearance of the scars over time (51%). Further, 68% stated that ZCL is dangerous.

Concerning perceptions on the causes of ZCL, the respondents mentioned the human responsibility for the environmental changes. The majority considered that ZCL is not hereditary (80%). It was not related to the quality of food (92%), or stress in everyday life (78%). However, 73% linked the environment (stagnant water) and physical activity (working alongside animals) with the incidence of ZCL. About 66% thought extreme temperatures contribute to the incidence of ZCL. Close to three quarters (73%) saw a correlation between stagnant water, sand flies and the incidence of ZCL. About fifty percent made a link between ZCL and abundant rainfall that frequently ends by forming stagnant water. This may be because these events rarely occur in Sidi Bouzid. The summer is usually very hot and associated easily with sand flies and increased risk of sand fly bites and illness.

Almost 71% of the respondents mentioned the notion of vectors and reservoirs. Close to two thirds linked rat’s sand flies to ZCL transmission. Yet, barely 43% said that killing rats and sand flies vectors is relevant to preventing risk of exposure to ZCL.

Eighty five per cent of the respondents thought that sand flies are responsible for the disease. Eighty seven per cent admitted there is a correlation between working with animals and exposure to ZCL. None of the subjects believed that the sandfly that are related to ZCL are small and almost invisible. Seventy per cent said that the sand fly that are linked to ZCL are the same size as other mosquitoes.

We found that the respondents’ perceptions on the time it takes for scars to develop ranged from an uncertain period (some scars are permanent while others appear and then fade away) to chronicity, sometimes with an increase in the number and appearance of the scars. Almost half the number of respondents stated that the scars do not fade quickly, but they were carefully optimistic that the scars would fade gradually with time.

The respondents said unanimously that ZCL has had no positive effects on their lives. The consequences have rather been negative. About 73% suffered social exclusion and stigmatization. Their relationships have been broken and they face more interpersonal conflicts in society, regardless of the context (family, social or professional). The consequences were seen also in their chances of getting employment. ZCL affected women who stay at home more than those who study or are already working. It reduced marriage prospects for men (75%) and women (59%). The consequences were felt also on aesthetic features, for the scars alter women’s beauty (58%).

With respect to individual ability to control ZCL, the most common perception was an absolute lack of power to control and cure the disease. The respondents thought there is nothing useful they can do to control the disease, its symptoms or its progress. Drugs were seen to have little or no effect, and the risk of getting the disease again is still likely.

When it comes to understanding the problem, we noted that patients still cannot explain how they contracted ZCL. There is little understanding of the problem, and respondents keep asking themselves "why am I the one who is infected".

We found that the emotional representations associated with ZCL were associated with the loss of self-esteem, feelings of inferiority and the idea that the disease is equal to an obvious social disadvantage. There was also a strong sense of shame.

Table 2 summarizes the relationship between illness perception and sociodemographic and clinical factors. The correlation analysis shows that high education level and high knowledge levels are associated with greater cognitive sensitivity (perception of consequences) and emotional sensitivity (emotional representations). Hence, variables such as "age" and "education level" have to be monitored closely, for they may partly explain why there is a variance in the IPQ-R results.

**Table 2:** Relationship between illness perception, age, level of education, date of ZCL and number of scars.

	Identity	Chronicity	Causes	Consequences	Curability	Controllability	Coherence	Emotional Representation
**Age**	0.12	0.23	- 0.03	- 0.28	- 0.05	0.15	- 0.01	**- 0.32**
	p = 0.441	p = 0.137	p = 0.829	p = 0.076	p = 0.742	p = 0.329	p = 0.922	**p = 0.041**
**Level of education**	- 0.05	- 0.19	0.07	**0.33**	- 0.03	- 0.11	0.17	**0.33**
	p = 0.717	p = 0.227	p = 0.648	**p = 0.040**	p = 0.818	p = 0.495	p = 0,280	**p = 0.032**
**Total knowledgescore**	0.13	- 0.08	0.04	0.18	- 0.16	0.11	**0.44**	0.19
	p = 0.432	p = 0.616	p = 0.764	p = 0.267	p = 0.323	p = 0.528	**p = 0.004**	p = 0.228
**ZCL date**	0.05	0.13	- 0.07	- 0.07	0.13	0.15	- 0.07	- 0.24
	p = 0.744	p = 0.423	p = 0.646	p = 0.661	p = 0.406	p = 0.328	p = 0.662	p = 0.134
**Number of facial scars**	- 0.01	- 0.17	0.12	- 0.08	0.09	- 0.05	0.24	- 0.02
	p = 0.929	p = 0.275	p = 0.432	p = 0.582	p = 0.575	p = 0.719	p = 0.133	p = 0.855
**Number of body scars**	0.02	- 0.02	0.01	0.02	0.26	0.13	- 0.06	0.13
	p = 0.898	p = 0.865	p = 0.978	p = 0.860	p = 0.095	p = 0.407	p = 0.672	p = 0.397
**Total number of scars**	0.01	- 0.11	0.06	- 0.01	0.31	0.11	0.04	0.12
	p = 0.933	p = 0.508	p = 0.702	p = 0.937	p = 0.058	p = 0.521	p = 0.793	p = 0.459

The inter-correlation matrix for multiple IPQ-R domains (Table 3) showed that the domains do not all measure the same constructs, because not all of them are inter-correlated. The matrix showed further that the "identity" domain has a negative correlation with the "curability" domain (r = -0.35, p<0.05) and a positive correlation with the "coherence" domain (r = 0.41, p<0.01). These results suggest that the more patients can clearly identity ZCL, the better they will perceive it coherently and the more they will know it is incurable. We noted also the presence of a correlation (r = -0.30, p = 0.048) between the "cause" domain and the "curability" domain: it seems that the more patients know about CL, the more pessimistic they get about the prospects of recovering from the disease.

**Table 3:** Correlation matrix for multiple IPQ-R domains.

	Identity	Chronicity	Causes	Consequences	Curability	Controllability	Coherence
**Chronicity**	- 0.04						
	p = 0.815						
**Causes**	0.23	- 0.17					
	p = 0.149	p = 0.276					
**Consequences**	0.24	- 0.18	- 0.11				
	p = 0.138	p = 0.266	p = 0.476				
**Curability**	**-0.35**	0.19	- 0.30	- 0.15			
	**p = 0.027**	p = 0.231	p = 0.048	p = 0.363			
**Controllability**	- 0.05	- 0.08	- 0.04	- 0.04	0.16		
	p = 0.744	p = 0.625	p = 0.782	p = 0.802	p = 0.303		
**Coherence**	**0.41**	- 0.29	0.01	**0.53**	- 0.15	0.01	
	**p = 0.008**	p = 0.066	p = 0.934	**p = 0.000**	p = 0.339	p = 0.941	
**Emotional representation**	0.24	- 0.09	- 0.09	**0.77**	- 0.15	0.01	**0.48**
	p = 0.132	p = 0.557	p = 0.558	**p = 0.000**	p = 0.338	p = 0.936	**p = 0.001**

This correlation analysis suggested also that patients who have a more coherent perception of ZCL do have stronger emotional reactions (r = 0.48, p<0.001) and face more severe consequences (r = 0.53, p<0.001). Finally, those who are more inclined to perceive ZCL as an illness with negative consequences were more likely to claim that they experience severe emotional difficulties (r = 0.77, p<0.001).

All these significant correlations are maintained even after testing them on the "age" variable and the "education level" variable, using partial correlations.

### Stress and stigma among women with facial scars caused by ZCL

The descriptive data focused more on anticipation of a negative reaction from others and the efforts made to display anticipatory avoidance behaviour meant to deny such reactions, than on convictions from the presence of negative experiences that patients have had through social interactions on the state of their skin (Table 4).

**Table 4:** Relationship between illness perception, stress, and stigma.

	Identity	Chronicity	Causes	Consequences	Curability	Controllability	Coherence	Emotional Representation
**Anticipation and avoidance of stress**	0.02	- 0.17	- 0.11	**0.34**	- 0.05	0.03	**0.31**	**0.42**
	p = 0.882	p = 0.302	p = 0.514	**p = 0.031**	p = 0.720	p = 0.818	**p = 0.050**	**p = 0.007**
**Rejection experience**	0.02	- 0.18	0.12	0.19	0.08	0.03	0.11	0.19
	p = 0.901	p = 0.262	p = 0.452	p = 0.220	p = 0.621	p = 0.816	p = 0.493	p = 0.236
**Total Stress**	0.01	- 0.22	- 0.06	**0.29**	- 0.01	0.11	**0.29**	**0.34**
	p = 0.991	p = 0.168	p = 0.712	**p = 0.068**	p = 0.975	p = 0.513	**p = 0.069**	**p = 0.032**

"Rejection experiences" and the "anticipation and avoidance of stress" were respectively, significantly and negatively correlated with age (r = -0.33, p<0.05 and r = -0.31, p<0.05). The younger the subject, the higher the number of experiences tied to stigma; and the older the subject, the more such feelings are dismissed. Thus, the correlation analysis showed that stigma assessment indices were not correlated with level of education, "total knowledge" score, or time of illness. They were correlated only with age: the younger the subject, the more they tend to experience stigma.

Furthermore, there are moderate correlations and just around the significant level between the total number of body scars and "rejection experiences" (r = 0.31, p<0.05), and between the number of facial scars and the scores on "Anticipation and avoidance of stress" (r = 0.29, p = 0.06). It is important to note that this correlation between the number of scars and stigma indices was at the same threshold of significance when checked against age-related variations. Hence, the number of body scars had a strong link to experience with stigma, regardless of a patient’s age.

We also noted correlations between the scores on multiple IPQ-R domains (identity, chronicity, causes, consequences, curability, controllability, coherence and emotional representations) and PLSI factors (rejection experience and the anticipation and avoidance of stress.

The results showed that anticipation and avoidance of stress is strongly correlated with the scores on "Consequences", "Coherence" and "Emotional representation." These results suggest that the more a person looks at the world anticipating segregation and rejection, the more they perceive ZCL as a disease with harmful effects on them, the more they see it as a mysterious and incomprehensible illness, and the more they talk about suffering and emotional difficulties. Emotional representation also appeared to be significantly correlated with the total score on stress, as seen in the PLSI.

### Impact of ZCL on quality of life

**Fig 2:**
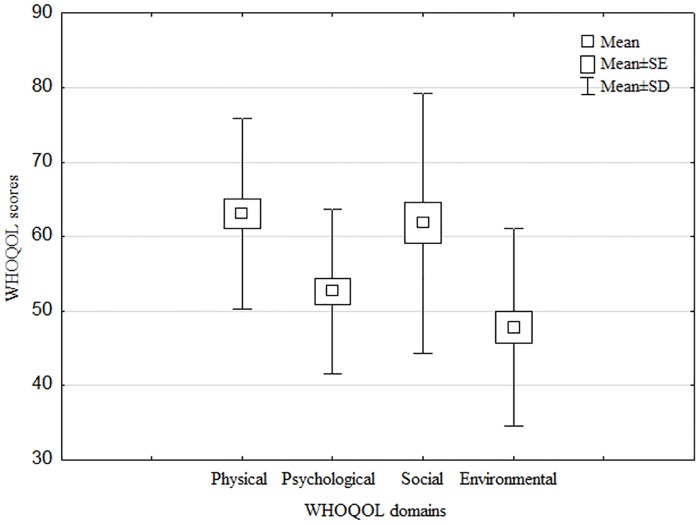
Levels of quality of life perceived in the Whoqol-26.

The scores on quality of life of women with ZCL facial scars showed that various domains of quality of life are perceived in a very different manner (p<0.001). Environmental quality of life is seen as significantly less fulfilling (p<0.001) than quality of social life. According to the respondents, it appeared to be far less fulfilling than (p<0.001) the physical quality of life. Quality of mental life did not differ much from environmental quality of life. However, it is said to be significantly more impaired than quality of social life (p<0.001) and the physical quality of life (p<0.001). The physical quality of life was not described as significantly different from quality of social life (Fig 2).

Quality of life scores were not significantly correlated with either age, education level, time of illness, or the number of facial or body scars (Table 5). In addition, the correlations between quality of life scores and the multiple IPQ-R domains were all insignificant.

**Table 5:** Relationship between quality of life and demographic and clinical data.

	Age	Education level	Date of ZCL	Nb of facial scars	Nb of body scars	Total nb. of scars	Total knowledge
**Physique QOL**	-0.29	0.22	-0.08	0.05	-0.05	-0.03	0.15
	p = 0.069	p = 0.165	p = 0.625	p = 0.757	p = 0.739	p = 0.854	p = 0.347
**Mental QOL**	-0.03	0.07	- 0.01	0.12	- 0.12	- 0.06	0.14
	p = 0.832	p = 0.680	p = 0.956	p = 0.458	p = 0.454	p = 0.697	p = 0.387
**Social QOL**	-0.01	-0.15	0.11	- 0.18	0.20	0.11	**-0.34**
	p = 0.971	p = 0.361	p = 0.507	p = 0.270	p = 0.213	p = 0.481	**p = 0.030**
**Environmental QOL**	-0.21	0.10	- 0.13	0.18	0.13	0.21	- 0.16
	p = 0.200	p = 0.533	p = 0.428	p = 0.270	p = 0.435	p = 0.204	p = 0.316

**Fig 3:**
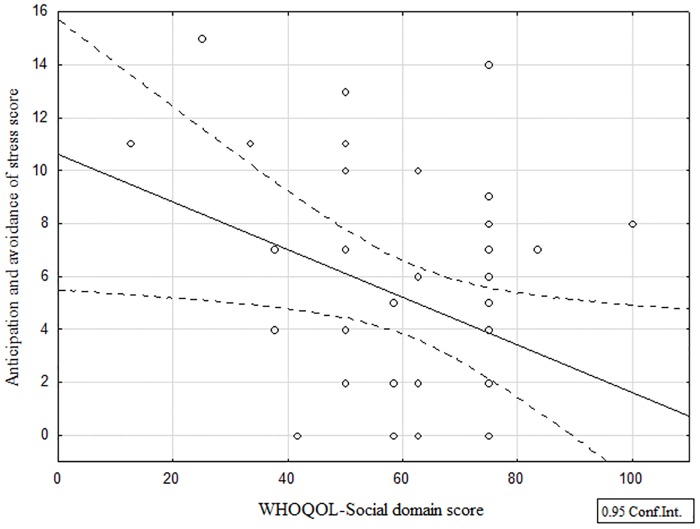
Relationships between quality of social life and anticipation and avoidance of stress score.

**Fig 4:**
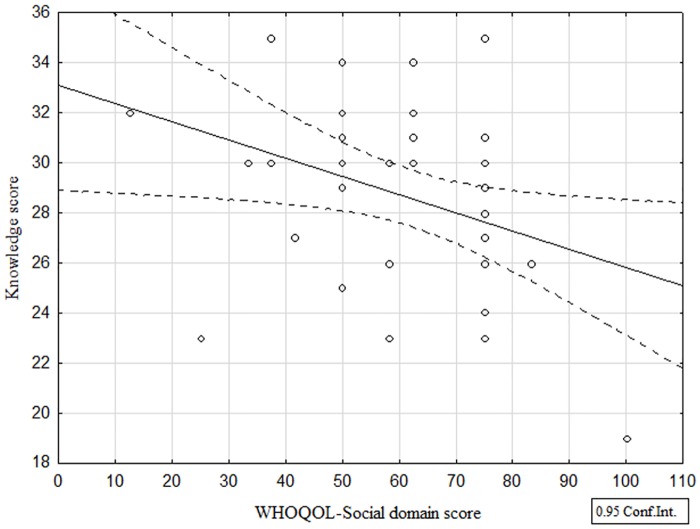
Relationship between quality of social life and ZCL knowledge score.

On the other hand, quality of life scores had interesting correlations with PLSI scores on stigma (Figs 3 and 4). The quality of social life score was significantly and negatively correlated to the "Anticipation and avoidance of stress" score (r = -0.36, p<0.05) suggesting that the more a person anticipates segregation and psychosocial stress, the lower their quality of social life was seen to be. Equivalent correlations were observed with the "total stress" score (r = -0.32, p<0.05): the more a person experiences high stress levels in society, the lower the quality of their social life was seen to be.

Finally, there was a significant and negative correlation between the score on quality of social life and that on "Total Knowledge". It seems that the higher a person’s knowledge of ZCL, the less likely they are to view their quality of social life in a negative manner (r = -0.34, p<0.05). The quality of knowledge seems therefore to be tied closely to the quality of social life (Table 6).

**Table 6:** Relationship between quality of life and psychosocial adjustment (PLSI).

		Anticipation avoidance of stress	Rejection experience	Total Stress
**WHO quality of life (WHOQOL 26) scale**	**Physical QOL**	- 0.13	- 0.11	- 0.14
		p = 0.431	p = 0.528	p = 0.389
	**Mental QOL**	- 0.26	**- 0.27**	**- 0.27**
		p = 0.104	**p = 0.092**	**p = 0.087**
	**Social QOL**	**- 0.36**	- 0.06	**- 0.32**
		**p = 0.025**	p = 0.730	**p = 0.047**
	**Environmental QOL**	- 0.13	0.01	- 0.16
		p = 0.434	p = 0.980	p = 0.311

It was important also to note that the correlation between scores on 'Rejection experience" and on mental quality of life index was on the verge of the threshold of significance (r = -0.27, p<0.092). Mental quality of life tends to be perceived negatively, if the subject claims to have suffered many rejection experiences. The mental quality of life index tended to be associated with the total stress and stigma score (r = -0.27, p<0.087).

## Discussion

The objective of this study is to explore the perceptions of ZCL, the stigma associated with the disease, and the quality of life of people with ZCL in Sidi Bouzid.

We studied the perceptions of CL based on the theoretical model by Leventhal et al. [4], which identifies the factors involved in patients’ formation of cognitive representations of their illness. Research on the observation of treatment showed that the reasons for adopting health-enhancing behaviours depend on the representational and cognitive factors related to their state of health [8].

It is the first time this type of model, and the assessment tool associated with it (IPQ-R), is applied to study CL. For example, on the 18^th^ of February, 2016, we found that **ScienceDirect** contained **1 758** articles on "IPQ-R" and **21 831** articles on "leishmania", but no entry on "IPQ-R" and "leishmania".

It is the first time also that the psychological and psychosocial aspects of CL are studied in the Arab world, which is one of the areas with the highest number of cases of CL in the world. All works dealt basically with the epidemiological and/or biological aspects of the issue. Despite the originality of this work, our results should be taken with caution and have to be confirmed because we only did an explanatory study on a small sample size.

Based on the results from the IPQ-R, we see that patients tend to consider ZCL as a disease that affects entire generations and is more rampant in some regions more than others. The disease mostly affects women and spreads in cyclical trends, caused by "mosquito" bites associated with the presence of rats, and related to climate change and the favourable ecological context in Sidi Bouzid. The patients were incline to think ZCL has negative consequences on their personal, family, social and professional life. They generally believe that ZCL considerably alters their natural beauty and reduces their prospects of getting employment, getting married and being valued in society. They see ZCL as a mysterious, incurable and uncontrollable disease.

After analysing the responses we had gathered under the “causes” domain in the survey questionnaire, we were able to identify patients’ beliefs on the underlying causes of ZCL. The contents relate basically to biological and/or ecological causes. The patients did not talk about irrational, supernatural and magical causes, as was the case with some patients in Colombia [9].

ZCL patients think that living with the disease is a steady source of stress that forces them to develop anticipatory avoidance behaviour against rejection. They try to anticipate stigma primarily out of feelings of inferiority in society. These problems bring about a huge sense of injustice and anger to the point where there is little sympathy with other girls or women with ZCL. These difficulties can lead to depression and anxiety. The patients think their scars are degrading and this increases their feelings of shame. They describe their feeling of isolation, that is, they think subjectively that they are inferior to other members of society, that they are not fully fledged individuals, or that they have an "impaired identity" and suffer discrimination, which includes rejection and isolation from others. Anticipated stigma is a subjective experience that could negatively affect psychosocial and professional development and adjustment among patients. These results largely confirm the findings from similar studies conducted on patients with vitiligo [10].

The psychological effects of ZCL tend to reduce as patients get older. The more educated a patient is, the more his knowledge of the disease develops, and the more he tends to analyse the consequences of the disease and display dramatic emotional reactions. A high level of education and high levels of knowledge are associated with greater cognitive sensitivity (perception of consequences) and emotional sensitivity (emotional representations).

After calculating our correlation analysis, the results we obtained were consistent with the theoretical construct on illness perception in Leventhal’s model [4]. We noted a positive and significant correlation between the "consequences", "emotional representation" and "coherence" domains. The more the patient with CL considers that the disease is mysterious and incomprehensible, the more severe would be its consequences and effects on their emotional life are seen to be. The ability to identify the disease has a significant and negative correlation with the ability to cure it. It suggests that the more a patient is able to identify CL, the lower their hope that it can be cured. These correlations are strong and are not attenuated by controlling the level of education.

Moreover, there is a high level of consistency between perceptions of CL in the IPQ-R and stigma indices in the PLSI. Anticipation and avoidance of stress have a strong correlation with a patient’s perceptions of the "consequences", "coherence" and "emotional representation" of the disease. The more patients see ZCL as a disease with severe, unambiguous consequences that have harmful emotional effects, the more they tend to expect to be rejected by others, and the more they would be inclined to form avoidance attitudes towards others. These conclusions do not concern the relationships between these variables and the patient’s age, level of education, or knowledge of the disease.

The correlation between the level of stigma and the number of lesions is not related to the location of these lesions on the face or elsewhere on the body. This rather surprising result confirms the work of Papadopoulos et al. [10] who report that the location of vitiligo does not play a critical role in the way patients experience stigma.

Our results also suggest that the nature of stigma that patients experience are associated with certain general fears and anticipation of rejection, rather than the possible rejection that patient truly experience from society. However, this is not the case in some countries like Pakistan, where Afghan refugees with CL face stigma and are excluded from social groups [1], or in Afghanistan where women with CL are separated from their children because of fears that the mothers will contaminate the children [2]. Stigma that results from the disfiguring scars of CL apparently depends on how patients think others perceive them. This means that the disability associated eventually with the disfigurement depends on how patients see themselves in their own environment (personal, social and professional).

The respondents believe that ZCL has a generally negative impact on quality of life. But our results show that ZCL has differential impacts on the four domains of quality of life in the WHOQOL-26. The quality of life of ZCL patients is largely unsatisfactory in two domains: environmental quality of life and quality of mental life. The respondents are inclined to think that the environmental quality of life is the more degraded of the two. It includes availability of financial resources, freedom, security, access to and quality of housing, opportunities for acquiring new information and skills, opportunities for recreation and leisure, as well as the availability and quality of transport facilities. We are assuming that ZCL patients are not the only ones dissatisfied with this domain. The entire population shares this dissatisfaction with the environmental quality of life in the region of Sidi Bouzid.

For the respondents, the quality of mental life is lower than physical quality of life and the quality of social life. It suggests that they are dissatisfied with their self-image and appearance, with the negative sentiment displayed towards them, with their self-esteem and their cognitive abilities (the ability to think, learn, memorize and concentrate). This latter result is frequently described in literature on skin infections [10].

None of the domains on quality of life is closely correlated with the scores that reflect patients’ perception of ZCL in the IPQ-R. However, the quality of social life domain is significantly and negatively correlated to the "Anticipation and avoidance of stress" factor in the Psoriasis Life Stress Inventory (PLSI) questionnaire. The more a person expects to face rejection and stigma, the lower the quality of social life they have when it comes to personal relations and social support. These dynamics have been observed already in patients with psoriasis [11].

ZCL got little attention for long because, apart from the aesthetic effects it has, the disease is neither contagious nor fatal, and causes no major health concerns. However, it can have adverse socio-economic effects. ZCL is a health problem in developing countries because of the social and economic challenges it causes [12]. The disease leaves disfiguring scars on the face or any other part of the body bitten by mosquitoes. It undermines the social roles and social identity of patients, reduces their chances of being accepted by others or getting employment in institutions, and induces psychological distress.

These results show that public health officials, in planning control programmes, should pay attention to the gaps in knowledge perceptions, the vulnerability of women to cutaneous leishmaniasis, the ways of preventing their exposure and the psychological support to reduce the psychosocial effects [13].

With the Leventhal model [4], we have assessed the mental perceptions of ZCL in patients in Sidi Bouzid and what they make of this problem. Understanding these personal perceptions of the disease is a key first step in efforts to seek help, design an adaptive strategy or adopt a management regime for the disease [14]. The studies that are going to be done on these therapeutic aspects should pay attention to these perceptions of CL, so that they put forward ideas on how to improve quality of life for patients. The work of Nilforoushzadeh et al. [15] shows that providing psychological care contributes effectively to improve the psychological and social well-being of these patients, and enhances their commitment to follow courses of treatment. Lusli et al [16,17] showed the benefit of psychosocial support like counselling on the leprosy-related stigma. Moreover, Peters et al [18,19] illustrated the success of a contact intervention in Indonesia to reduce leprosy-related stigma in the community. These interventions are likely to work for CL as well.

It is crucial also to take preventive measures, given the importance of behavioural aspects in the onset of the disease. Byrne et al. [20] have described cognitive and behavioural therapy that was done to change patients’ illness perceptions after a heart attack, and how this improved the quality of life for the patients. Ensuring that the causal model on ZCL places more emphasis on environmental factors may be the first step in behavioural therapy for this epidemic. This would mean creating opportunities for people concerned to personally control future trends of the disease and the risk of contamination through other insect bites in the future. Combating real and/or perceived stigmatization is another decisive step in the delivery of care, because stigma has negative impacts on the quality of life, mental health and participation in social and professional life.

## Supporting Information

S1 Table (XLSX)

S1 Checklist (DOC)
